# The Influence of Snacking on Glycaemia in Women With Gestational Diabetes Mellitus: A Systematic Review

**DOI:** 10.1111/mcn.70079

**Published:** 2025-07-28

**Authors:** Olivia Righton, Catherine V. George, Cathal O'Hara, Gráinne Kent, Lucilla Poston, Wendy L. Hall, Angela C. Flynn, Sara L. White

**Affiliations:** ^1^ Department of Nutritional Sciences School of Life Course and Population Sciences, King's College London London UK; ^2^ School of Population Health, Royal College of Surgeons in Ireland (RCSI) Dublin Ireland; ^3^ Department of Women & Children's Health School of Life Course and Population Sciences, King's College London London UK

**Keywords:** blood glucose, diabetes, female, gestational, glucose intolerance, glycemic control, pregnancy, snacks

## Abstract

Gestational diabetes mellitus (GDM) is characterised by glucose intolerance identified during pregnancy, typically resolving postpartum. Globally, the standardised prevalence is 14%. Dietary management is essential in mitigating adverse maternal and infant outcomes, with guidelines recommending low‐ or no‐carbohydrate snacks to stabilise blood glucose concentrations. However, the influence of snacking patterns on glycaemic outcomes in women with GDM has yet to be assessed by systematic review. Following PRISMA guidelines, this review examines the association between snacking patterns, specifically timing, frequency, and composition, and glycaemia in this population. Electronic searches were conducted in MEDLINE, Embase, Maternity and Infant Care (MIDIRS), and CINAHL in April 2024, with an updated search performed in June 2025. Reference lists and citations were also screened. Studies examining the impact of snacking on glycaemia in women with GDM were included. Of the 7405 articles identified, five studies met the inclusion criteria: four 1–8‐week intervention studies and one 7‐day observational study. The included studies showed considerable heterogeneity in their designs. Evidence for a relationship between snacking and glycaemia in GDM was mixed, with some studies suggesting glycaemic benefits from snacking, including bedtime snacking, while others found that bedtime snacks adversely influenced glycaemia. Any association between snacking and glycaemia in individuals with GDM remains inconclusive due to limited available data, study heterogeneity, and conflicting results. The lack of high‐quality studies underscores the need for further research to define the role of snacking in glycaemia among this population.

## Summary

- Women with GDM are encouraged to consume low‐ or no‐carbohydrate snacks to reduce fluctuations in blood glucose, but this guidance is limited by a lack of robust evidence.
- This review highlights the limited evidence on how snack timing, frequency, and composition influence glycaemia in women with GDM, with only five relevant studies identified.
- The only observational study suggested that snacking two to three times per day may improve glycaemia, but intervention trials have not varied or monitored daily snack frequency.
- Bedtime snacks showed no consistent glycaemic benefit and, in two studies, were associated with poorer fasting or postprandial glucose.
- Further research, including longer‐duration, randomised controlled trials with detailed dietary monitoring, is needed to clarify how snack timing, frequency, and composition impact glycaemia and inform dietary recommendations for managing GDM.

## 1 Introduction

Gestational diabetes mellitus (GDM) is characterised by glucose intolerance that is identified during pregnancy and typically resolves postpartum. The global prevalence of GDM ranges from 7.1% to 27.6%, with this wide variability influenced by race, ethnicity, age, body composition, screening, and diagnostic criteria (Wang et al. 2022). In 2021, the global standardised prevalence was estimated to be 14.2% (Wang et al. 2022), rising in parallel with rates of obesity, sedentary lifestyles, and advanced maternal age (Hu et al. 2021). A recent meta‐analysis of 156 cohorts involving over 7.5 million pregnancies found that GDM, when treated without insulin, was significantly associated with elevated risks of caesarean delivery (16%), preterm birth (51%), and large‐for‐gestational‐age neonates (57%) (Ye et al. 2022). GDM requiring insulin therapy was associated with more than a twofold increase in the risk of neonatal intensive care unit admission (Ye et al. 2022). Beyond pregnancy, women with GDM face a 20‐fold higher lifetime risk of developing Type 2 diabetes, with nearly half exhibiting impaired glucose metabolism within a decade (Saravanan et al. 2020).

Attaining optimal glycaemia is critical for mitigating or preventing adverse maternal and infant outcomes, and diet plays a pivotal role in GDM management. For some women, dietary modifications can adequately modulate postprandial blood glucose (PBG) concentrations, thereby obviating the need for pharmacological interventions such as insulin therapy (Brown et al. 2017). In a UK cohort of 762 women with GDM, 32% achieved adequate glycaemia through lifestyle modification alone (Martine‐Edith et al. 2021). Obesity, smoking, and elevated fasting glucose were associated with a higher likelihood of requiring medication.

Current UK and US guidelines recommend that women with GDM maintain stable blood glucose concentrations through regular, evenly distributed meals and 2–3 snacks, favouring a selection of nutrient‐dense foods and avoiding those containing added sugars and high‐glycaemic index (high‐GI) carbohydrates, with total carbohydrate intake comprising approximately 35%–45% of daily energy intake (American Diabetes Association 2024; Diabetes UK 2023; National Institute for Health and Care Excellence 2022). These recommendations are based on evidence from systematic reviews (e.g., Brown et al. 2017), evidence grading, and expert consensus. Snacking may aid glycaemic regulation in GDM by helping to prevent postprandial glucose spikes through redistribution of carbohydrate load throughout the day. The British Dietetic Association provides detailed recommendations regarding snacking, advising that women with GDM incorporate low‐ or no‐carbohydrate snacks into their diets to reduce fluctuations in blood glucose (Beckwith et al. 2024), while the National Health Service recommends that snacks for women with GDM contain no more than 10–15 g of carbohydrates (NHS Diabetes Services for Adults, Maternity Services, & Royal Free London NHS Foundation Trust 2023). However, robust evidence guiding optimal snack timing, frequency, and composition in the management of GDM is lacking.

Large and recent datasets are scarce, but evidence suggests that snacking is prevalent among pregnant women. In the US Pregnancy, Infection, and Nutrition (PIN) study (*n* = 2065), over 70% of participants reported consuming food five or more times daily during the second trimester (Maria Siega‐Riz et al. 2001). A more recent study in Ireland (*n* = 565) found that pregnant women with overweight or obesity shifted from a meal‐dominant eating pattern at 16 weeks of gestation to a snack‐dominant pattern by 28 weeks, likely influenced by physiological changes, fluctuations in appetite, nausea, fatigue, and routine disruptions (Ainscough et al. 2020).

Contrasting findings from South Korea (*n* = 263) demonstrated a stable snacking frequency from preconception to mid‐pregnancy in both pregnant women with GDM and those with normal glucose tolerance. However, women with GDM were more likely to snack at night (Park et al. 2013). In non‐pregnant populations, bedtime snacking has been linked to poorer glycaemic outcomes (Bermingham et al. 2024; Kant and Graubard 2015; Xiaoyang et al. 2017), although such findings may not directly apply to women with GDM due to distinct metabolic adaptations (Zhu et al. 2021). Dietitians and diabetes educators may even recommend a bedtime snack for some women with GDM to prevent nocturnal hypoglycaemia, reduce hepatic glucose output, and manage fasting glycaemia (Francois et al. 2021).

Evidence on snack composition during pregnancy reveals a disconnect between dietary guidelines and actual intake among women with diabetes. Although women with Type 1 diabetes represent a distinct hyperglycaemic population from those with GDM, their dietary patterns may offer relevant insights. In a UK and Ireland study of 93 pregnant women with Type 1 diabetes, the mean energy and carbohydrate intakes met guideline targets; however, 46% of the carbohydrate intake came from non‐recommended sources, such as sugary drinks and confectionery, with one‐third of these consumed as snacks (Neoh et al. 2020). Similarly, a South Korean study of women with GDM found that while fruit was the most common snack (34.4%), it was followed by biscuits, bread, and dairy (Park et al. 2013). While this may indicate a high intake of lower‐quality carbohydrates, such conclusions depend on the nutritional quality of the bread and dairy products consumed. Although limited in number, existing studies suggest that pregnant women with diabetes may frequently consume snacks high in added sugars and refined carbohydrates, potentially impacting glycaemia.

Given that snacks can substantially contribute to carbohydrate intake, and considering the progressive insulin resistance, impaired glucose tolerance, and circadian dysregulation in GDM (Zhu et al. 2021), it is essential to understand the relationship between snacking and glycaemia in women with GDM. To inform dietary management recommendations, this systematic review examines reported associations between snacking patterns and glycaemic outcomes in women with GDM, focusing on three key aspects: (1) frequency, (2) timing, and (3) composition of snacks.

## 2 Methods

This systematic review was reported in accordance with the Preferred Reporting Items for Systematic Reviews and Meta‐Analyses (PRISMA) guidelines (Page et al. 2021) (Supporting Information S1: Figure S1) and the Synthesis Without Meta‐Analysis (SWiM) reporting guidelines (Campbell et al. 2020). It was registered in the PROSPERO database, with registration number CRD42024515710.

### 2.1 Literature Search

A search strategy was developed with input from an information specialist at King's College London, UK, to identify studies relevant to women with GDM, snacking, and glycaemia. The electronic databases MEDLINE, Embase, Maternity and Infant Care (MIDIRS), and CINAHL were initially searched in April 2024, with a repeat search conducted in June 2025 (Supporting Information S1: Figure S2). Searches were restricted to studies involving humans and published in English or with an existing English translation. There were no restrictions based on the year of publication.

### 2.2 Inclusion and Exclusion Criteria

The inclusion and exclusion criteria were developed using a PICOST (population, intervention/exposure, comparator/control, outcomes, study design, timeframe) structure (Sobue et al. 2021) (Table 1).

**Table 1:** The population intervention comparison outcome study design timeline (PICOST) eligibility criteria.

Parameter	Description
Population	Women diagnosed with GDM at any gestational age
Intervention/exposure	Exposure to snacking (foods or drinks containing calories consumed between main meals) – any composition, frequency, and timing
Comparator/control	Other women with GDM that consumed a different snack or no snack
Outcomes	Primary: Glycaemic parameters including but not limited to FBG, PBG, nocturnal glucose, daytime glucose, and HbA1C Additional: Metabolic function (fatty acids, amino acids, glycolysis‐related metabolites, ketone bodies and inflammatory markers), insulin, insulin resistance
Study design	Quantitative studies with any study design (cohort, cross‐sectional, case‐control, randomised and non‐randomised controlled trials) mixed methods
Timeframe	No restrictions on the publication date

Abbreviations: FBG = fasting blood glucose, PBG = postprandial blood glucose.

For inclusion, studies had to meet the following criteria: (1) quantitative or mixed methods studies that assessed snacking (defined as foods or drinks containing calories consumed between main meals; any composition, frequency, and timing) in women with GDM at any gestational age; (2) data reporting glycaemic outcomes including, but not limited to, fasting blood glucose (FBG) concentration, PBG concentration, nocturnal glucose, daytime glucose, and HbA1C. Studies that met the following criteria were excluded: (1) qualitative studies, systematic and narrative reviews, editorials, conference abstracts, policy papers, guidelines, and grey literature; (2) women with pre‐existing diabetes (Type 1 or Type 2 diabetes); (3) data reported on glycaemic outcomes not related to snacking; (4) studies not published in English.

### 2.3 Selection of Studies and Data Extraction

Records from each database search were imported into Rayyan (Ouzzani et al. 2016) for de‐duplication and initial screening. The titles and abstracts were screened independently and in duplicate. One reviewer (OR) reviewed all the records, while three other reviewers (CG, GK, CO'H) reviewed a subset. Any disagreements among reviewers were resolved by consensus. Eligibility was assessed based on the PICOST inclusion and exclusion criteria.

**Figure 1:**
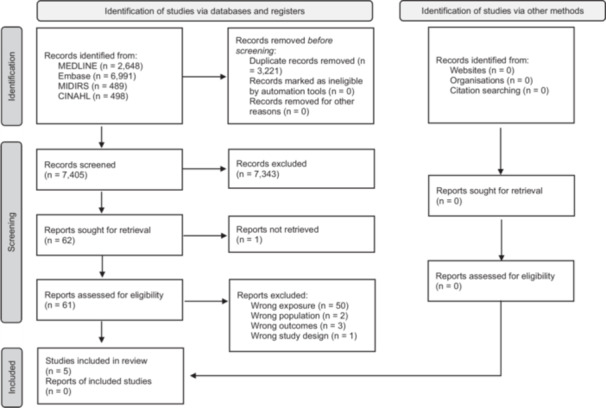
PRISMA flow diagram. n = number of individuals.

Full‐text articles were retrieved and reviewed for studies that met the inclusion criteria. One reviewer (OR) reviewed all full texts, and three reviewers (CG, GK, CO'H) reviewed one‐third each. Disagreements during the full‐text screening were resolved through discussion among the reviewers. Reference lists of included studies were screened, and citation searches using Google Scholar were conducted to identify studies citing the included studies. The results of the screening and selection process were documented using a PRISMA flow chart (Figure 1).

One reviewer (OR) performed data extraction, validated by a second reviewer (CG, GK, or CO'H). The characteristics and findings of the eligible studies were entered into standardised tables and checked for completeness and accuracy. The extracted data included the study title, authors, publication date, country, study type, objectives, population characteristics, inclusion and exclusion criteria, snacking data, glycaemic outcomes, and other relevant outcomes outlined in Table 2.

**Table 2:** Study characteristics.

Author, year	Title	Country	Study aims	Study design	Eligibility criteria	Sample size	Sample characteristics	GDM criteria
Feng et al. (2021)	Whey Protein Preloading Can Alleviate Stress Adaptation Disorder and Improve Hyperglycaemia in Women with GDM	China	To investigate the effect of whey protein preloading on stress hormones and PBG concentration in women with GDM.	Non‐randomised controlled trial	Inclusion: (1) Singleton pregnancy; (2) FBG < 6.1 mmol/L before pregnancy. Exclusion: (1) Known medical complications e.g., Types 1 and 2 diabetes, PCOS, thyroid disorders; (2) Treatment with hormones or drugs affecting blood glucose.	*N* = 60	Intervention group: Maternal age: 25.9 ± 3.1 years, Pre‐gestational BMI: 24.0 ± 3.4 kg/m^2^, Gestational weeks: 25.2 ± 2.4. Control group: Maternal age: 26.5 ± 2.3 years, Pre‐gestational BMI: 23.5 ± 2.7 kg/m², Gestational weeks: 26.4 ± 1.8. No significant differences in these characteristics between groups.	American Diabetes Association
Henze et al. (2022)	The Effect of Bedtime Snacks on FBG concentration in GDM	Australia	To investigate how various bedtime snacks (high‐carb, low‐carb, or none) affect FBG concentration in women with diet‐controlled GDM.	Prospective randomised crossover trial (participants completed three 5‐day Interventions)	Inclusion: (1) Aged 18+; (2) Singleton pregnancy (24–34 weeks); (3) Consistent diet patterns; (4) > 2 FBG between 4.7 and 5.4 mmol/L; (5) Received diabetes education and diet review; (6) Willing to perform FBG between 6 AM and 8 AM with at least 8 h since bedtime snack; (7) Evening meal between 5 PM and 8 PM; (8) Using Accu‐Chek or OneTouch Verio metres with accuracy within specified limits. Exclusion: (1) Required insulin or other oral diabetes therapies; (2) Shift worker or irregular mealtimes; (3) > 4 fasting BGLs ≥ 5.4 mmol/L; (4) Macrosomic fetus or polyhydramnios; (5) Required interpreting service; (6) Prior bariatric surgery; (7) Administered betamethasone or required hospital admission during study.	*N* = 68	Median maternal age of whole sample: 34 years (IQR: 31–37), Median BMI: 29.4 kg/m² (IQR: 27.0–34.6), Median gestation at recruitment: 30.8 weeks (IQR: 28.9–32.0), Mean FBG before intervention: 4.8 mmol/L (SD 0.45) Ethnicity: 61.2% Caucasian, 32.8% Asian, 6% other.	International Association of the Diabetes and Pregnancy Study Groups (IADPSG)
Nas and Yılmaz (2023)	The effect of fruit consumption time on glucose regulation in pregnancy with GDM	Turkey	To investigate the effect of fruit consumption time on FBG and PBG concentrations in women with GDM.	Non‐randomised controlled trial	Inclusion: (1) Singleton pregnancy; (2) No prior diagnosis of diabetes mellitus, impaired glucose tolerance, or hypoglycaemia before pregnancy; (3) No diagnosis of GDM in previous pregnancies; (4) Absence of comorbid diseases; (5) Pre‐pregnancy BMI between 20–25 kg/m²; (6) Not using insulin; (7) Maximum weight gain of 10 kg during pregnancy; (8) No conditions preventing fruit consumption; (9) Diagnosed with GDM based on OGTT results (24–28 weeks). Exclusion: (1) Multiple pregnancy; (2) Not consuming fruit; (3) Additional diseases other than GDM (e.g., Types 1 and 2 diabetes, impaired glucose tolerance, hypoglycaemia, preeclampsia, hypertension, kidney diseases); (4) Using insulin.	*N* = 64	Pre‐pregnancy BMI: Group 1: 25.31 ± 4.15, Group 2: 26.52 ± 4.22. No significant differences in age, education level, employment status, family history of diabetes, drug use, physical activity level, smoking habit before pregnancy, anthropometric measurements (height, BMI, current body weight, weight gain during pregnancy) between groups (*p* > 0.05). However, a difference was found between the groups in terms of smoking habit during pregnancy and having a history of food allergies (*p* < 0.05).	American Diabetes Association
Zhu et al. (2025)	The effect of bedtime snacks on morning fasting blood glucose in gestational diabetes mellitus: a randomised controlled trial	China	To evaluate whether bedtime snacking (25 g nuts) prevents high fasting blood glucose in women with GDM and its effects on glucose and lipid metabolism in late pregnancy.	Randomised controlled trial (open‐label, parallel‐group)	Inclusion: Pregnant women ≥ 18 years, diagnosed with GDM at 24–28 weeks, no bedtime snack habit before study. Exclusion: Pre‐existing diabetes, cardiovascular disease, thyroid disorders, nut allergy, cognitive or visual impairment, or current participation in other studies.	*N* = 62	Intervention group (*n* = 31): mean age 29.39 ± 3.54 years; Control group (*n* = 31): mean age 31.42 ± 3.80 years; Overall BMI: 28.02 ± 7.39 kg/m²; 98.4% participants Han ethnicity. No significant differences in baseline BMI or most lifestyle characteristics between groups.	IADPSG
**Observational**								
Morris et al. (2019)	Relationship of the frequency, distribution, and content of meals/Snacks to Glycaemic Control in GDM: The myfood24 GDM Pilot Study	United Kingdom	To examine nutritional intakes in women with GDM using the myfood24 tool, focusing on meal and snack frequency, daily distribution of calories and carbohydrates, and their impact on FBG and PBG concentrations.	Prospective observational pilot study (online 24‐h dietary recalls)	Exclusion: (1) Unable to read and understand English; (2) Prescribed medication for GDM.	*N* = 199	Mean maternal age: 33.3 ± 5.0 years Mean BMI: 29.7 ± 6.5 kg/m² (*n* = 196) Ethnicity: 57.3% White, 27.1% Asian, 10.1% Black, 5.5% Mixed, Chinese, or Other Parity: 36.7% Primiparous, 63.3% Multiparous	National Institute for Health and Care Excellence (NICE)

Abbreviations: BMI = body mass index, FBG = fasting blood glucose, IQR = interquartile range, PBG = postprandial blood glucose.

### 2.4 Study Quality

The methodological quality of each eligible study was assessed using the Effective Public Health Practice Project (EPHPP) Quality Assessment Tool for Quantitative Studies (Jackson and Waters 2005). One reviewer (OR) appraised all eligible studies, with a second reviewer (CG, GK, or CO'H) validating each appraisal. Each study received an overall rating of ‘strong’, ‘moderate,’ or ‘weak’ based on evaluations across eight categories: selection bias, study design, confounders, blinding, data collection methods, withdrawals and dropouts, intervention integrity, and analyses. In cases of inconsistent assessments, discrepancies were resolved by consensus.

### 2.5 Analysis

The tabulated study characteristics were scrutinised for notable variations that could contribute to heterogeneity. Due to the diversity of interventions and the heterogeneous nature of the results, a meta‐analysis was not deemed appropriate. Therefore, a narrative synthesis was constructed for the included studies following the SWiM guidelines (Campbell et al. 2020) (Supporting Information S1: Figure S3). The data in this systematic review were presented in textual and tabular formats, offering a comprehensive summary of the findings.

## 3 Results

The study selection process is illustrated in Figure 1. From the 10,626 articles initially identified, 3221 duplicates were removed. After screening 7405 titles and abstracts, 62 full texts were assessed, with 57 excluded, primarily due to a lack of snacking‐specific data, as they focused on main meals. No additional studies were found through citation searching, resulting in five studies being included in the final review.

### 3.1 Study Characteristics

The characteristics of the included studies are summarised in Table 2. The studies were conducted between 2019 and 2025 and ranged in study design. Four of the included studies were interventional. Feng et al. (2021) conducted a 14‐day non‐randomised controlled trial investigating the impact of whey protein preloading (in liquid form) on PBG concentrations in women with diet‐controlled GDM in China. Henze et al. (2022) performed a 15‐day prospective randomised crossover trial examining how different bedtime snacks impact FBG concentrations in women with diet‐controlled GDM in Australia. Nas and Yılmaz (2023) conducted a 7‐day non‐randomised controlled trial investigating the impact of fruit consumption timing on FBG and PBG concentrations in women with GDM in Turkey. Zhu et al. (2025) conducted an 8‐week randomised controlled trial in China to evaluate the effects of a bedtime mixed nut snack on FBG and PBG concentrations in women with diet‐controlled GDM. Morris et al. (2019) conducted a 7‐day prospective observational pilot study in the UK, assessing nutritional intakes in women with diet‐controlled GDM using the myfood24 tool, focusing on meal and snack frequency, daily distribution of calories and carbohydrates, and their associations with FBG and PBG concentrations.

### 3.2 Participant Characteristics

The sample sizes of the included studies ranged from 60 to 199 participants. The reported average maternal age spanned from 25 to 34 years, while the average body mass index (BMI) at study entry ranged from 23 to 29.7 kg/m^2^. In the three studies that reported gestational age at recruitment, participants were enroled between 24 and 30 weeks of gestation (Feng et al. 2021; Henze et al. 2022; Zhu et al. 2025), corresponding to the late second and early third trimesters. Ethnicity data available in two studies indicated a predominance of white participants (57.3%–61.2%) (Henze et al. 2022; Morris et al. 2019), while in the study by Zhu et al. (2025), 98.4% were of Han Chinese ethnicity. Four studies exclusively enroled participants with singleton pregnancies and excluded women with a pre‐pregnancy diagnosis of Type 1 or Type 2 diabetes mellitus (Feng et al. 2021; Morris et al. 2019; Nas and Yılmaz 2023; Zhu et al. 2025). All five studies excluded women treated with insulin, and four excluded women using other oral diabetes therapies for managing GDM (Feng et al. 2021; Henze et al. 2022; Morris et al. 2019; Zhu et al. 2025). Additionally, four studies excluded participants with other medical complications or comorbidities, including polycystic ovary syndrome, thyroid disorders, preeclampsia, hypertension, and kidney diseases (Feng et al. 2021; Henze et al. 2022; Nas and Yılmaz 2023; Zhu et al. 2025).

### 3.3 Study Snack Characteristics

Snacking exposures varied in the included studies and are summarised in Table 3. Feng et al. (2021) assessed snack timing and composition. Their intervention group received a 250 mL drink containing 20 g of whey protein isolate as a preload 30 min before lunch for 14 days. The control group received a 250 mL preload drink with 20 g of control flavouring. This study was included as the preload aligned with this review's definition of snacking (Table 1). Snack timing and composition were also assessed by Henze et al. (2022), who used a randomised, counterbalanced design where participants completed three 5‐day interventions in a Latin Square Design. The interventions included (1) a higher‐carbohydrate bedtime snack consisting of a small tub of plain yoghurt and a small‐medium apple (providing 220 calories, 25 g carbohydrate, 10 g protein, 7.7 g fat, and 3.3 g fibre), (2) a lower‐carbohydrate bedtime snack of 10 almonds and 20 g of dark chocolate (providing 184 calories, 7.4 g carbohydrate, 4.2 g protein, 14.8 g fat, and 1.1 g fibre), and (3) no bedtime snack. Both snacks were low‐GI.

**Table 3:** Exposure characteristics.

Author, year	Snack composition	Snack timing	Snack frequency and study duration
Feng et al. (2021)	Intervention: 250 mL preload drink with 20 g whey protein isolate (73 kcal) Control: 250 mL preload drink with 20 g control flavouring (6.5 kcal)	30 min before lunch	1 per day for 14 days
Henze et al. (2022)	Higher carbohydrate snack: 1 small plain yogurt and 1 small to medium apple (220 kcal, 25 g carbohydrate, 10 g protein, 7.7 g fat, 3.3 g fibre) Lower carbohydrate snack: 10 almonds and 20 g dark chocolate (184 kcal, 7.4 g carbohydrate, 4.2 g protein, 14.8 g fat, 1.1 g fibre) No Snack	≥ 2 h after evening meal (evening meal between 5 PM and 8 PM)	1 per day for 5 days each, totalling 15 days (5 days for each of the 3 snacks)
Nas and Yılmaz (2023)	Fruits with low‐GI (e.g., apple, orange, mandarin) (200 g per day) accounting for 10% of daily carbohydrate intake	Group 1 consumed fruit with main meals and an alternative carbohydrate‐matched snack Group 2 consumed fruit as snacks	3 servings per day for 7 days
Zhu et al. (2025)	25 g of mixed nuts (walnuts, hazelnuts, Brazil nuts, pistachios, cashews, macadamias)	2 h after dinner and within 1 h before bedtime	1 snack per day for 8 weeks
**Observational**			
Morris et al. (2019)	Carbohydrate and calorie intakes of snacks and drinks (containing kcal) consumed between meals (% of daily intake)	Morning (12 AM–12 PM) Afternoon (12 PM–6 PM) Evening (6 PM–12 AM)	Fewer than 2 snacking periods a day; ≥ 2 and < 3 snacking periods a day; 3 snacking periods a day i.e., morning, afternoon and evening

Abbreviation: GI = glycaemic index.

In the non‐randomised trial by Naz and Yilmaz (2023), snack composition was assessed. One group consumed fruit with main meals, while the other consumed it as a snack for 7 days. Both groups adhered to an isocaloric diet, structured into seven daily eating episodes (three main meals and four snacks), with equal macronutrient and energy intake distribution. The group that did not consume fruit as snacks was given macronutrient‐ and energy‐matched snacks. The randomised controlled trial by Zhu et al. (2025) also assessed snack composition as well as snack timing. For 8 weeks, participants consumed a 25 g portion of mixed nuts (including walnuts, hazelnuts, Brazil nuts, pistachios, cashews, and macadamias) as a low‐carbohydrate, high‐fat, high‐protein snack daily, 2 h after dinner and 1 h before bedtime.

Morris et al. (2019) conducted the single observational study and assessed snack frequency and timing. The study asked participants to self‐record their dietary intake five times over the 2 weeks following diagnosis using the web‐based 24‐h dietary recall tool myfood24. This tool offers over 45,000 food and drink options and various portion‐size choices. Snacks were considered any food or drinks containing calories consumed in the morning between breakfast and lunch, in the afternoon between lunch and dinner, and in the evening after dinner.

### 3.4 Glycaemic Outcomes

All five studies measured FBG concentrations, and four reported PBG concentrations (Feng et al. 2021; Morris et al. 2019; Nas and Yılmaz 2023; Zhu et al. 2025) (Table 4). In the 14‐day intervention study conducted by Feng et al. (2021), FBG and PBG concentrations were measured on days 3, 5, 7, and 14. In the 15‐day intervention study by Henze et al. (2022), participants self‐recorded their FBG and PBG concentrations daily. Similarly, in the 7‐day intervention study by Naz and Yilmaz, participants self‐recorded their food intake along with FBG and PBG concentrations daily. In the 8‐week intervention study by Zhu et al. (2025), participants self‐recorded their FBG concentrations three times per week and their 1‐ and 2‐h PBG concentrations twice weekly. In the 7‐day observational study by Morris et al. (2019), participants self‐recorded their FBG and PBG concentrations 1 h after breakfast, lunch, and dinner for 7 days. None of the included studies assessed other glycaemic parameters relevant to this review, such as nocturnal glucose, daytime glucose, and HbA1C.

**Table 4:** Study outcomes.

Author, year	Glycaemic outcomes	Additional relevant outcomes	Study outcome measures	Study outcomes
Feng et al. (2021)	FBG 2‐h PBG	Insulin IR Oxidative stress	Blood glucose: Roche automatic biochemical analyser Insulin: Electrochemical luminescence immunoassay IR: HOMA‐IR formula Oxidative stress: MDA, SOD, and GSH assays	No difference between groups in FBG (*p* > 0.05). PBG decreased on days 3, 5, 7, and 14 in the intervention group compared to the control group (*p* < 0.05). Plasma 2‐h insulin increased on days 5, 7, and 14 in the intervention group (*p* < 0.05, 0.05, 0.01). HOMA‐IR decreased on day 14 in the intervention group (*p* < 0.05). In the intervention group, MDA decreased by 20.7% (*p* < 0.01) and SOD decreased by 13.4% (*p* < 0.05) on day 14 only, and GSH decreased by 16.7% and 29.1% on days 7 and 14, all different from the control group (*p* < 0.05).
Henze et al. (2022)	FBG	Requirement for insulin	Blood glucose: Accu‐Chek or OneTouch Verio home glucometers Requirement for insulin: assessed by treating physician	Both snacks resulted in higher FBG compared to no snack: Higher carbohydrate snack: Mean FBG 4.96 mmol/L (mean difference 0.09 mmol/L, 95% CI: 0.05–0.13, *p* < 0.001). Lower carbohydrate snack: Mean FBG 5.01 mmol/L (mean difference 0.14 mmol/L, 95% CI: 0.09–0.18, *p* < 0.001). Lower carbohydrate snack led to a FBG that was 0.05 mmol/L higher than the higher carbohydrate snack (95% CI: 0.002–0.09, *p* = 0.042). 4 participants withdrawn due to requiring insulin during study period
Nas and Yılmaz (2023)	FBG PBGs		Home glucometer	Decrease in PBG in both groups on day 7 (*p* < 0.05). No significant difference between groups. Decrease in PBG‐FBG difference on day 7 in both groups (*p* < 0.05). No significant difference in the change of PBG‐FPG difference between groups (*p* > 0.05).
Zhu et al. (2025)	FBG, 1‐ and 2‐h PBGs, incidence of morning hyperglycaemia	Lipid profile (LDL, HDL, TG, TC), HbA1c, birth outcomes (birth weight, gestational age, delivery mode, macrosomia, preterm birth)	FBG and PBG: Home glucometer (3×/week for FBG, 2×/week for PBG); Lipids and HbA1c: lab testing at 34 weeks; birth outcomes: hospital records	No significant difference in average FBG (4.90 vs. 4.96 mmol/L, *p* = 0.720) or hyper‐FBG incidence (0.19 vs. 0.26, *p* = 0.335). LDL significantly higher in snack group (3.21 vs. 2.52 mmol/L, *p* = 0.031). Higher incidence of hyper 1h‐PBG in snack group (0.42 vs. 0.28, *p* = 0.036). No significant differences in HbA1c, TC, TG, HDL, or perinatal outcomes between groups.
**Observational**				
Morris et al. (2019)	FBG 1‐h PBGs		Self‐recorded capillary blood glucose measurements taken 4 times daily for 7 days: FBG before breakfast, and then 1‐h PBGs after breakfast, lunch, and dinner.	18% of daily kcal came from snacks and drinks, including those consumed in the morning, afternoon, and evening between meals. Those achieving FBG and PBG targets (≤ 5.3 mmol/L and < 7.8 mmol/L, respectively) more likely to snack ≥ 2 per day (*p* = 0.008). Only 8.3% (*n* = 10) of participants snacked 3 times per day; 9/10 of them met the FBG target. 70.6%–77.2% of snacks consumed by women achieving FBG and PBG targets contained less than 20 g carbohydrates. 76.6%–86.4% of snacks consumed by women not achieving FBG and PBG targets contained less than 20 g carbohydrates. Between groups *p* > 0.05. Participants who achieved FBG target consumed a higher % of their daily kcal (6% vs. 3.5%; *p* = 0.03) and carbohydrates (6.5% vs. 3.3%; *p* = 0.031) from evening snacks and drinks compared to those who exceeded the target. Total kcal (33.4% vs. 36.1%) and carbohydrate intake (30.3% vs. 33.5%) from evening meals were similar between those achieving the FBG target and those exceeding it.

Abbreviations: FBG = fasting blood glucose, GSH = glutathione, IR = insulin resistance, MDA = malondialdehyde, PBG = postprandial blood glucose, SOD = superoxide dismutase.

The intervention studies yielded mixed results. Henze et al. (2022) found that high‐ and low‐carbohydrate evening snacks elevated FBG concentrations compared to no evening snack. The high‐carbohydrate snack increased mean FBG by 0.09 mmol/L (95% CI: 0.05–0.13, *p* < 0.001) compared to the no‐snack option, while the low‐carbohydrate snack increased FBG by 0.14 mmol/L (95% CI: 0.09–0.18, *p* < 0.001). The increase in FBG following the low versus the high carbohydrate snack (0.05 mmol/L) achieved significance (95% CI: 0.002–0.09, *p* = 0.042).

Feng et al. (2021) reported no significant difference in FBG concentrations between the intervention group, who consumed a 250 mL whey protein drink as a snack and the control group (*p* > 0.05). However, the intervention group exhibited a significant reduction in 2‐h PBG concentrations on days 3, 5, 7, and 14 compared to the control group (*p* < 0.05).

Naz and Yilmaz (2023) observed a significant reduction in PBG concentration by day 7 in both the group that consumed fruit as a snack and the group that consumed a macronutrient‐ and energy‐matched snack (*p* < 0.05), with no significant difference between the groups. Additionally, the difference between PBG and FBG (PBG‐FBG) was lower in both groups by day 7 (*p* < 0.05) but was not different between groups.

Zhu et al. (2025) found no significant difference in mean FBG concentration (4.96 vs. 4.90 mmol/L; *p* = 0.720) or the incidence of morning hyperglycaemia (0.26 vs. 0.19; *p* = 0.335) between the intervention and control groups. However, a significantly higher incidence of elevated 1‐h PBG was observed in the snack group (0.42 vs. 0.28; *p* = 0.036), with no significant differences in 2‐h PBG concentrations. Zhu et al. (2025) also assessed HbA1c at 34 weeks of gestation, and no significant differences were reported (*p* > 0.05).

Morris et al. (2019) reported that snacks and calorie‐containing drinks consumed between meals accounted for 18% of daily energy intake. They found a significant positive association between women who regularly consumed snacks ( ≥ 2 per day) and their ability to achieve FBG targets of ≤ 5.3 mmol/L and 1‐h PBG targets of < 7.8 mmol/L (*p* = 0.008). Most snacks consumed by participants, regardless of whether they achieved FBG and PBG targets, contained less than 20 g of carbohydrates. Additionally, women who met the FBG target consumed a higher percentage of their daily energy (6% vs. 3.5%; *p* = 0.03) and carbohydrates (6.5% vs. 3.3%; *p* = 0.01) from evening snacks and drinks compared to those who exceeded the target.

### 3.5 Additional Outcomes

Two studies measured insulin requirements during the study period (Feng et al. 2021; Henze et al. 2022), while one study assessed insulin resistance (Feng et al. 2021) (Table 4). Feng et al. (2021) reported a significant increase in plasma 2‐h insulin levels in the intervention group consuming the whey protein snack on days 5, 7, and 14 (all *p* < 0.05). Additionally, a significant reduction in HOMA‐IR (Homeostatic Model Assessment of Insulin Resistance) was observed in the intervention group on day 14 (*p* < 0.05). Henze et al. (2022) measured insulin requirements to exclude participants retrospectively. Four participants required insulin during the study period, and their data were consequently excluded from the results. Zhu et al. (2025) monitored the use of insulin and oral hypoglycaemic agents via a follow‐up questionnaire, and two participants who reported usage were excluded from sensitivity analyses.

Other metabolic function outcomes relevant to this review were not routinely measured. However, Feng et al. (2021) assessed oxidative stress markers, finding that malondialdehyde (MDA) levels in the intervention group decreased by 20.7% (*p* < 0.01), while superoxide dismutase (SOD) levels reduced by 13.4% on day 14 only (*p* < 0.05), and glutathione (GSH) levels decreased by 16.7% and 29.1% on days 7 and 14, respectively, all showing significant differences from the control group (*p* < 0.05). Moreover, Zhu et al. (2025) assessed lipid profiles (LDL, HDL, triglycerides, and total cholesterol) at 34 weeks’ gestation. A significantly higher LDL concentration was observed in the snack group compared to the control group (3.21 vs. 2.52 mmol/L; *p* = 0.031), while no significant differences were reported for other lipid markers. Only the study by Nas and Yılmaz (2023) assessed weight gain during the intervention and reported no significant differences between the groups (*p* = 0.120).

### 3.6 Quality Appraisal

All five studies were assessed for methodological quality. Four studies were rated as ‘weak’ in quality (Feng et al. 2021; Henze et al. 2022; Morris et al. 2019; Nas and Yılmaz 2023). In contrast, the study by Zhu et al. (2025) received a ‘moderate’ rating due to its randomised design, control for confounders, and high retention rate. Due to the nature of dietary interventions, blinding participants and investigators was impossible in the four intervention studies (Feng et al. 2021; Henze et al. 2022; Nas and Yılmaz 2023; Zhu et al. 2025). The most common methodological concern was selection bias. For instance, three studies did not describe their sample selection process, including whether participants were randomly selected, systematically referred, or self‐referred (Feng et al. 2021; Henze et al. 2022; Nas and Yılmaz 2023) (Supporting Information S1: Figure S4). Another notable weakness in the evidence was that only two intervention studies randomised participants to treatment (Henze et al. 2022; Zhu et al. 2025).

## 4 Discussion

This systematic review examined how snack frequency, timing, and composition relate to glycaemia in women with GDM. Five studies were identified: four intervention studies (three short‐term interventions of 7–15 days and one longer‐term intervention of 8 weeks) and one 7‐day observational study. High heterogeneity was observed, particularly in snack type and study design.

The available data on snack frequency and glycaemia were limited. The pilot observational study by Morris et al. (2019) indicated that regular snacking was associated with improved glycaemia. Those achieving FBG and PBG targets were more likely to snack two to three times daily than those who snacked less frequently. In the four intervention studies, both intervention and control groups received snacks at the same frequency, which prevented direct comparisons. While two intervention studies included a no‐snack control (Henze et al. 2022; Zhu et al. 2025), neither study tracked participants’ consumption of additional snacks outside the intervention, which prevented an accurate assessment of total snack frequency and its relationship with glycaemia.

Evidence on snack timing and glycaemia was also limited and inconsistent. Morris et al. (2019) identified a positive association between consuming a higher proportion of daily energy and carbohydrates from evening snacks and drinks and meeting the FBG target. Conversely, Henze et al. (2022) and Zhu et al. (2025) reported that bedtime snacks increased FBG and PBG concentrations, respectively. These contradictory findings were echoed in a recent systematic review of 16 studies (*n* = 374) on bedtime snacking in non‐pregnant individuals with Type 2 diabetes, which found no consistent association between bedtime snacking and improved glycaemia, especially when compared to a no‐snack control group (Roach et al. 2022). The diverse results between the studies of Morris et al. (2019), Henze et al. (2022) and Zhu et al. (2025) may stem from differences in definitions of snack timing; the former defined evening snacks broadly as any food or drink containing calories consumed after dinner, whereas the latter two studies required participants to wait at least 2 h after dinner to have their bedtime snack, likely resulting in considerable variation in timing between the observational and intervention studies. Additionally, differences in cohort characteristics and underlying GDM physiology may further explain the contrasting findings (Francois et al. 2021).

Evidence on snack composition and glycaemia was also limited, but suggested that macronutrient composition may influence glycaemia more than carbohydrate content alone. Morris et al. (2019) found that most snacks contained less than 20 g of carbohydrates; however, no clear association emerged between carbohydrate load and achieving FBG and PBG targets. The number of snacks containing less than 15 g of carbohydrates, the recommended limit for women with GDM (NHS Diabetes Services for Adults, Maternity Services, & Royal Free London NHS Foundation Trust 2023), was not reported, preventing the assessment of lower‐carbohydrate snacks (≤ 15 g) on glycaemia. Henze et al. (2022) found that a lower‐carbohydrate (7.4 g) snack increased FBG more than a higher‐carbohydrate (25 g) protein‐rich snack (10 g vs. 4.2 g protein), suggesting an important role for protein in moderating the glycaemic response. Notably, protein is known to slow carbohydrate breakdown to glucose, reduce glucose absorption and stimulate insulin release (Yanagisawa 2023). Feng et al. (2021) also demonstrated improved PBG concentrations and plasma 2‐h insulin levels with a protein‐rich snack.

The findings of Nas and Yılmaz (2023) that both low‐GI fruit and a macronutrient‐matched snack improved PBG concentrations and the PBG‐FBG difference suggest that the overall macronutrient composition matters more than the specific food type in influencing glycaemic outcomes. However, it is important to note that none of the included studies assessed the food matrix of snacks, such as dietary fibre content, despite its well‐established role in modulating postprandial glycaemia by slowing gastric emptying and digestion (Goff et al. 2018), which highlights a gap in the literature.

### 4.1 Strengths and Limitations

The strengths of this review include a comprehensive literature search across four electronic databases, supplemented by reference and citation tracking, to maximise the identification of eligible studies. The screening process was conducted in duplicate, ensuring thoroughness, with data extraction and quality assessment initially performed by one reviewer and validated by a second reviewer. The review adhered to PRISMA guidelines, ensuring transparency, and utilised the EPHPP Quality Assessment Tool for Quantitative Studies, providing a consistent and objective evaluation. Additionally, the SWiM guidelines were followed for reporting the narrative synthesis, ensuring a clear and transparent presentation of the findings.

There were also several limitations. A meta‐analysis was not feasible due to substantial heterogeneity in study designs, snack types, and outcome measures, which limited the ability to quantify the average effects of snack timing, frequency, or composition on glycaemia and draw definitive conclusions. Secondly, restricting inclusion to English‐language studies may have introduced language bias by excluding relevant research. Furthermore, the small number of included studies reduces the generalisability of the findings and highlights the need for more high‐quality research.

Only one intervention study (Nas and Yılmaz 2023) measured daily energy and macronutrient intake, which limits the ability to isolate the effects of snack interventions in the other included studies, as differences in overall energy or nutrient intake may have influenced outcomes. For instance, in the study by Feng et al. (2021), the whey protein snack drink (containing 20 g whey protein isolate and 73 calories) may have increased satiety, subsequently affecting lunch intake and influencing PBG concentrations. Similarly, the studies by Henze et al. (2022) and Zhu et al. (2025) did not account for the macronutrient composition and energy intake of the evening meal, which may have impacted FBG and PBG measurements.

### 4.2 Recommendations

Future research should include longer duration, randomised controlled trials that vary snack frequency and monitor total daily snack intake to clarify their effects on glycaemia in women with GDM. Furthermore, more intervention studies should investigate the impact of bedtime snacks, particularly their timing and composition, on FBG concentrations. Healthcare providers may recommend that women with GDM consume a snack before bed (Francois et al. 2021); however, the current evidence on whether this practice is beneficial or counterproductive is conflicting.

Furthermore, interventions should assess lower‐carbohydrate snacks and consider their complete nutritional profile, including fibre content, to ascertain how the food matrix affects glycaemia. In future trials, detailed dietary monitoring is essential to control for total energy and macronutrient intake and to isolate the effects of snacking patterns on glycaemia.

## 5 Conclusion

To our knowledge, this review is the first to systematically evaluate the relationship between snacking and glycaemia in women with GDM. Evidence remains limited and inconsistent: while some studies suggest benefits from regular or protein‐rich snacks on glycaemia, two interventions found that bedtime snacks increased glucose concentrations compared to no‐snack controls. The heterogeneity of study designs, snack types, and inadequate dietary monitoring make it challenging to draw definitive conclusions. These findings highlight the necessity for well‐designed, longer‐term randomised controlled trials with detailed dietary assessment to inform evidence‐based snacking guidelines for GDM management.

## Author Contributions

O.R. and A.F. conceptualised the study. O.R. designed and executed the search strategy. O.R., C.G., C.O'H., and G.K. conducted the screening, data extraction, and data validation. O.R. drafted the original manuscript, which was reviewed and edited by A.F. and S.W. All authors have read and approved the final manuscript.

## Conflicts of Interest

The authors declared no conflicts of interest.

## Supporting information


**Figure S1:** PRISMA statement. **Figure S2:** Full search strategy for databases. **Figure S3:** SWiM reporting items. **Figure S4:** Quality assessment for included studies.

## Data Availability

The authors have nothing to report.
